# Immunomodulatory Effects of Vitamin D Supplementation in a Deficient Population

**DOI:** 10.3390/ijms22095041

**Published:** 2021-05-10

**Authors:** Mathieu Garand, Mohammed Toufiq, Parul Singh, Susie Shih Yin Huang, Sara Tomei, Rebecca Mathew, Valentina Mattei, Mariam Al Wakeel, Elham Sharif, Souhaila Al Khodor

**Affiliations:** 1 Research Department, Sidra Medicine, Doha 26999, Qatar; mtoufiq@sidra.org (M.T.); psingh@sidra.org (P.S.); shuang@sidra.org (S.S.Y.H.); stomei@sidra.org (S.T.); rmathew1@sidra.org (R.M.); valentinamattei3@gmail.com (V.M.); 2 Department of Biomedical Sciences, College of Health Sciences, Qatar University, Doha 26999, Qatar; mariamalwakeel94@gmail.com

**Keywords:** transcriptomic, 25-hydroxyvitamin D, 25(OH)D, immune system, immune response, vitamin D deficiency, Qatar

## Abstract

In addition to its canonical functions, vitamin D has been proposed to be an important mediator of the immune system. Despite ample sunshine, vitamin D deficiency is prevalent (>80%) in the Middle East, resulting in a high rate of supplementation. However, the underlying molecular mechanisms of the specific regimen prescribed and the potential factors affecting an individual’s response to vitamin D supplementation are not well characterized. Our objective is to describe the changes in the blood transcriptome and explore the potential mechanisms associated with vitamin D3 supplementation in one hundred vitamin D-deficient women who were given a weekly oral dose (50,000 IU) of vitamin D3 for three months. A high-throughput targeted PCR, composed of 264 genes representing the important blood transcriptomic fingerprints of health and disease states, was performed on pre and post-supplementation blood samples to profile the molecular response to vitamin D3. We identified 54 differentially expressed genes that were strongly modulated by vitamin D3 supplementation. Network analyses showed significant changes in the immune-related pathways such as TLR4/CD14 and IFN receptors, and catabolic processes related to NF-kB, which were subsequently confirmed by gene ontology enrichment analyses. We proposed a model for vitamin D3 response based on the expression changes of molecules involved in the receptor-mediated intra-cellular signaling pathways and the ensuing predicted effects on cytokine production. Overall, vitamin D3 has a strong effect on the immune system, G-coupled protein receptor signaling, and the ubiquitin system. We highlighted the major molecular changes and biological processes induced by vitamin D3, which will help to further investigate the effectiveness of vitamin D3 supplementation among individuals in the Middle East as well as other regions.

## 1. Introduction

Vitamin D is well recognized for its functions in the homeostasis of calcium and bone mineralization [1]. In many Arab countries, including Qatar, a high prevalence of vitamin D deficiency is observed despite ample sunshine [2]. Women cover most of their skin for cultural reasons and are therefore especially affected [3,4,5,6]. Additionally, a high prevalence of vitamin D deficiency was reported among college-age women in Qatar [7]. Thus, vitamin D deficiency is a national interest to Qatar and needs to be better understood. Vitamin D supplementation (D2 or D3) is usually prescribed to treat those with suboptimal levels; however, the response to supplementation vary widely among individuals—with some individuals demonstrating virtually no absorption of oral D2/D3 [8,9,10]. The reason for the failure of oral vitamin D regimens to effectively replete serum 25(OH)D level is likely multifactorial. Body weight (34.5%), type of supplement used (D2 or D3, 9.8%), age (3.7%), calcium intake (2.4%), and baseline 25(OH)D concentrations (1.9%) of the individuals can all contribute to the variations in post supplementation response and serum 25(OH)D levels (12). Genetic factors have also been proposed as a potential contributor [11] to responsiveness to vitamin D supplementation. Our current work has shown that gut microbiome composition may also play a role in the individual response to vitamin D supplementation [12].

Aside from its conventional health functions, there has been growing interest in the role of vitamin D as an immune modulator [13]. Reduced vitamin D levels have been associated with development of many autoimmune disorders such as multiple sclerosis [14], rheumatoid arthritis [15], systemic lupus erythematosus [16], and inflammatory bowel disease [17]. Vitamin D supplementation has been shown to mitigate the incidence and adverse outcomes of these diseases [18]. Taking vitamin D has also been associated with protection against respiratory diseases, especially in those who are very deficient (<10 ng/mL) [19]. Vitamin D insufficiency and deficiency was recently associated with increased respiratory mortality in a 15-years longitudinal cohort study with 9548 participants [20]. The putative implications of vitamin D3 supplementation for the prevention of, or as adjuvant to, respiratory diseases have been the subject of recent reviews and investigations [21,22]. It was recently proposed that vitamin D deficiency is associated with an increased risk of contracting or developing the symptoms of SARS-CoV-2 (COVID-19), speculatively by the development of an exaggerated inflammatory response [23,24]. However, results from other studies yielded contradicting conclusions [25], highlighting the need for further studies between vitamin D and COVID-19. Similarly, the specific immunoregulatory mechanisms affecting an individual’s response to vitamin D3 are also unclear.

There are numerous alternative pathways that can metabolize vitamin D to bioactive compounds with diverse effects. A disbalance among these alternative pathways can contribute to the development of diseases, and this topic has been recently reviewed in the context of cancer progression [26]. Thus, there are possibilities/risks for dysregulation of vitamin D functions in those with low 25(OH)D levels and/or those who do not respond to conventional supplementation regimens. In order to better tailor vitamin D treatment in high-risk group/individual (i.e., akin to an individualized treatment approach), it is critical to understand the molecular mechanisms by which vitamin D supplementation varies among individuals. To gain a better understanding of the molecular response to vitamin D under deficiency, we performed high-throughput PCR to profile the genes expression changes in whole blood obtained from a cohort of vitamin D-deficient, but otherwise healthy, participants who had undergone a weekly oral vitamin D3 supplementations (50,000 IU of 25(OH)D) for three months.

## 2. Results

### 2.1. Cohort Description

Of the final cohort of 80 participants, the majority were of Arab descent (70; 87.5%) and the average age was 21, with a range from 17–28 years old. The average BMI was 24.39, with 52 participants (65%) categorized as normal, 24 (30%) as overweight or obese, and 4 (5%) as underweight. Sixty-two participants (77.5%) had less than 1 h of average daily exposure to sun, and the majority (76%) of the cohort had a history of vitamin D deficiency. Sun exposure was not correlated to the overall gene expression (Figure S1). Participants had blood drawn at pre and post 25(OH)D (hereinafter referred to vitamin D3) supplementation, which consisted of a weekly dose of 50,000 IU vitamin D3 for three months. At the end of the intervention, participants were classified as either responder (R) (those who achieved vitamin D3 above 20 ng/mL) or non-responder (NR) (those whose vitamin D3 remained <20 ng/mL) (Figure 4A). Vitamin D levels among our participants prior to supplementation ranged from 2.5 to 22.8 ng/mL with an average value of 11 ng/mL (Figure 4B). Post-supplementation, the levels ranged from 2.96 to 62.72 ng/mL with an average value of 34.02 ng/mL (Figure 4C). The majority of the participants (70) fell into the R group. The remaining 10 participants were categorized in the NR group, in which nine were at levels well below 20 ng/mL (Figure 4C, black bars) and with one at 19.36 ng/mL (falling into a mix bin which is represented by a blue bar with black grid pattern).

### 2.2. Gene Expression Profiles

Our primary objective was to investigate the molecular changes in response to the regimen of vitamin D3 supplementation using a high-throughput PCR-based Transcriptomic Fingerprint Assay (TFA) (32) in whole blood taken from each participants pre and post supplementation. In brief, the TFA is composed of co-expression gene modules derived from 16 blood transcriptomic profiles in health and disease states (see Methods). Two samples were excluded from analysis due to poor PCR amplification signal (Figure 4D). An overview of the normalized expression pattern (dCt) of the final study samples (n = 160) for the TFA panel (264 genes) is expressed as a Euclidean heatmap (Figure 5A). Genes as rows were subjected to hierarchical clustering and columns (samples) were segregated by pre- (Phase I) and post-vitamin D3 supplementation (Phase II). Distinct and contrasting patterns of gene expression were observed between the two phases, indicating a clear gene expression modulation by vitamin D3 in all individuals.

We then performed principal component analysis (PCA) on the overall gene expression between the two phases and observed a separation across the first two components (Figure 5B). As a secondary objective, we compared the gene expression profile of responding groups (either R or NR) in order to gain insights about factors affecting the response to vitamin D3 (data not shown). However, the results showed no distinct separations of gene expression between the response groups within each phase. This suggests that the genes contributing to the differential expression profiles between the phases alone do not explain the repletion of circulating 25(OH)D levels in some participants compared with others.

To interpret the molecular effects of vitamin D3, we determined the DEGs, expressed as ddCT, between the two phases. We identified 54 significantly DEGs using the following criteria: Abs(fold-changes) > 2 and adjusted *p*-value < 3 × 10^−5^, determined by a Wilcoxon Signed Rank test. Interestingly, 98% (53 out of 54) of the significant DEGs exhibited a decrease in transcript abundance (Figure 5C; Supplementary Tables S1 and S2). Notably, our results showed that E4F1, a key mediator of cell growth, was the only gene significantly upregulated (2.4 FC). Downregulation of E4F1 has been shown to cause cell cycle arrest via the increases in ROS and DNA damage (38). Along the axis of cellular functions, supplementation induced the downregulations of MAP3K5 and MAP3K12 (−2.4 and −2.1 FC, respectively), which are involved in the mitogen-activated protein (MAP) kinases pathway and JNK/p38 cascade-dependent inflammatory cytokines production. MAP3K5 has also been shown to be essential for mediating innate immune responses from TNF or LPS (39). G protein-coupled receptors (GPCR) are known to mediate the cellular response to LL-37, an important antimicrobial peptide modulated by vitamin D3 (40). The gene coding for LL-37, cathelicidin antimicrobial peptide (CAMP), was not measured in our assay, however, multiple components of the GPCR signaling pathway were assessed. Genes involved in GPCRs (GNG2; −2 FC) and the conversion of PIP3 to PIP2 pathways (INPP5D, PIP4K2B aka PIP5K2B, PIK3CG; −2.4, −2.2, −2.6, respectively) were found to be down-regulated with vitamin D3 supplementation.

**Figure 1:**
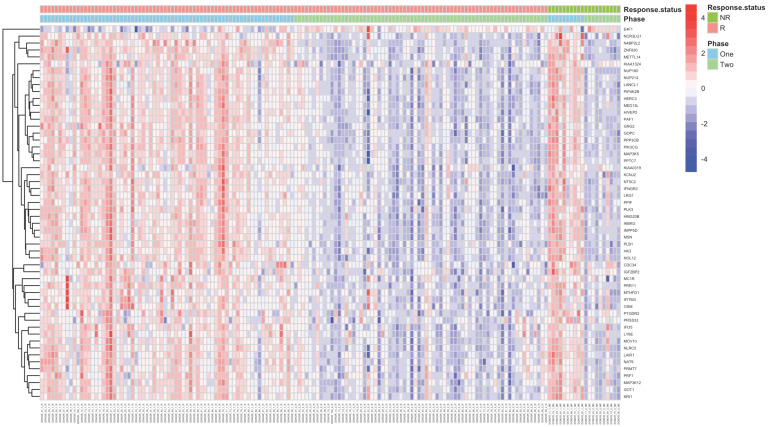
Drastic downregulation of gene expression observed with vitamin D3 supplementation. Normalized gene expression heatmap with unsupervised hierarchical clustering of significantly differentially expressed genes (n = 54) between supplementation periods and response group to supplementation. Each row represents a gene, and the columns represent the 80 study participants from the two supplementation phases/periods (total 160 columns).

We then compared the profiles of the 54 DEGs between the response groups (R and NR) and again observed mostly downregulation of gene expression by vitamin D3 in both groups; however, the response status did not majorly affect the overall gene expression pattern (Figure 1). We then examined the magnitude of the downregulation between the response groups and observed a downregulation trend that was consistently more pronounced in the NR group (Figure S2). Differential gene expression analysis revealed that the R group recapitulated most of the aforementioned DEGs (44 out of 54); while the low number of DEGs (3 out of 54) within the NR group (Phase II vs. I) are likely due to the small size of this subset (n = 10) (Supplementary Figures S3A–B).

### 2.3. Biological Processes Modulated by Vitamin D

**Figure 2:**
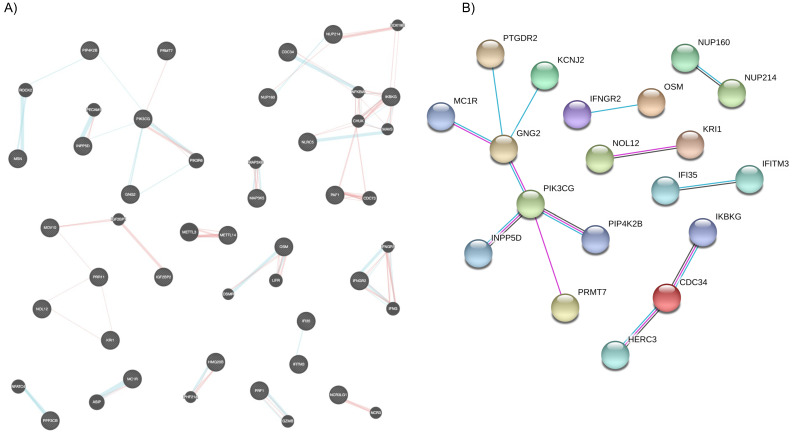
Key molecular processes are emphasized by network maps. (**A**) Network of relationships, weighted on biological process, among the differentially expressed genes (DEGs) induced by vitamin D3 (GeneMania). Edges denote physical interactions (orange) and pathways (blue). (**B**) Network of predicted protein–protein interactions inferred from the DEGs list (STRING). Edges shown depict known interactions based on knowledge from curated databases (blue edges), experimentally determined (pink edges), and co-expression data (black edges). A permalink is available for this network: https://version-11-0.string-db.org/cgi/network.pl?taskId=pagJWFiaVstn, accessed on 13 April 2021.

To investigate the biological relevance of the 54 DEGs in response to vitamin D3 supplementation, we generated a gene–gene network (GeneMania), focusing on “physical interactions” and “pathways” as parameters to define the associations (Figure 2A). We identified 13 clusters of two or more nodes with the two largest gene clusters centering on phospho-inositol-3 kinase (PI3K) signaling pathway and NF-kappa-B (NF-kB)/ubiquitin system.

We then generated a protein–protein interaction network (STRING; Figure 2B; https://version-11-0.string-db.org/cgi/network.pl?taskId=pagJWFiaVstn (accessed on 13 April 2021)) to gain further insights of the molecular relationships. The edges in the STRING network represent known protein interactions sourced from curated databases (blue edges), experimentally determined (pink edges), and co-expression data (black edges). We observed six clusters of two or more nodes, with PI3K pathway, again being the predominant cluster. The combined analyses showed prominent (57% of the biological processes) and significant changes in the immune system that involved immune responses, cytokine, and interferon signaling.

Modulation of cell surface receptor expression has implications for multiple intra-cellular signaling pathways and, in the case of vitamin D3, NF-kB is particularly important. We observed a significant reduction of NLRC5 expression (−2.6 FC), a regulator of the NF-kB and type I interferon signaling pathways (41,42), which suggests the enhancement of TNF/IL6 and type-1 IFN activation/sensitivity. However, discrepancies regarding the precise role of NLRC5 [27,28] in literature argues that a fine-tuned regulation may be exerted to subsets of cytokines and host response pathways. Additionally, decreases in CDC34 (−2 FC) and HERC3 (−2.1 FC), which are involved in polyubiquitination of NF-KBIA leading to its subsequent proteasomal degradation, as well as that of IKBKG (−2.6 FC; a subunit of the NF-KBIA phosphorylating complex), which is involved in the degradation of NF-KBIA, were also seen in this study. Collectively, these gene changes suggest an attenuation of NF-kB activity by vitamin D3 supplementation.

Interestingly, the observed reduction in IFNGR2 expression (−2.1 FC) may be linked to decreases in IFI35 and IFITM3 (−2.4 and −2.3 FC, respectively). The latter is involved in inhibiting cellular entry and replication to multiple virus such as flu and SARS-CoV (45). The reductions in nucleoporin molecules gene expression (NUP160 and NUP214; −2.1 and −2.1, respectively) also suggests a decrease in the import of substrates to nucleus and transactivation of gene expression. However, whether these nucleoporins are specific to pathways downstream of vitamin D3 remained unknown. In conjunction with the reported inhibition of MAP kinase phosphatases (MKP−1 and MKP−5) (43,44) and the expression of LL-37 (40), our results suggest that vitamin D3 plays a regulatory role on innate immunity via the downregulation of TLR4 pathway.

**Figure 3:**
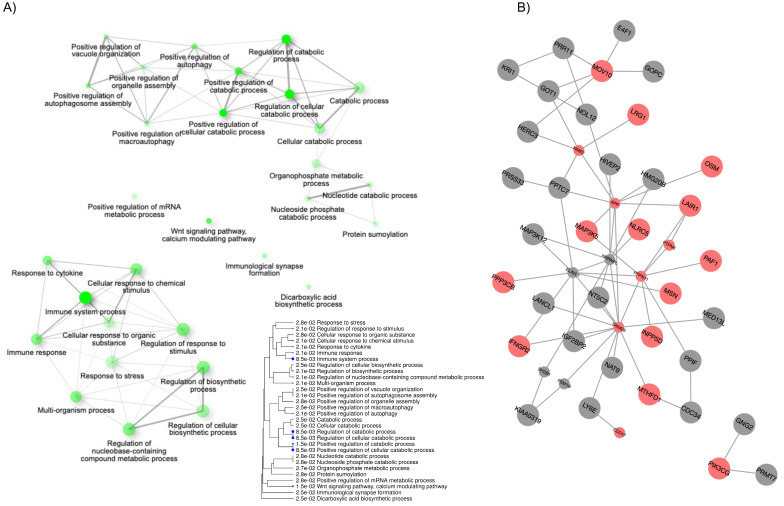
Gene set enrichment analyses reinforce the role of vitamin D3 on immune response modulation. (**A**) In the network map of gene ontology (GO) terms (top 30, ranked by enrichment FDR), connections are made if the terms (nodes) share 30% or more genes, with thicker edges represent more overlapped genes. Darker nodes reflect smaller p-values and size of the node reflects the number of genes. Gene composition of each term is available in Table S3. The term tree is a hierarchically ranked list with enrichment significance describing the hierarchical connection between GO terms. Larger blue dots indicate smaller p-values. The term tree and GO network were generated with ShinyGO v0.61. (**B**) Seeded network-based enrichment analysis was done using WebGestalt and identified 366 GO terms. The network at the top of the panel shows the seeded (i.e., input) gene names (large nodes), the top-ranking neighbor genes (small nodes), and the nodes involved in “immune system process” (GO:0002376) are highlighted in red.

In order to maximize the annotation coverage and to provide a more systematic interpretations for the DEGs, we performed gene set enrichment analyses using two methods [29,30]: Over representation test (ORT) via ShinyGo v0.61 [31] and seeded network-based enrichment analysis via WebGestalt R-based web tools [32]. ORT revealed that the top five most significant gene ontology (GO) terms encompassed immune system process, regulation of catabolic process, and Wnt and calcium modulating pathways (Figure 3A); for the full list of enriched GO terms and corresponding gene composition, see (Table S3). The GO network depicts the relationship between GO terms at a cutoff of ≥30% gene overlaps. Thicker edges represent more overlapping genes between GO terms. Darker nodes represent enrichments with smaller p-values while the size of the nodes reflects the number of genes. Based on the similarity among the gene composition of the enriched GO terms, we identified two major clusters representing: (1) Cellular responses to stimuli, such as cytokine, involving the immune system, and (2) cellular catabolic processes involving intracellular vesicles, in response to vitamin D3.

The seeded network-based enrichment analysis utilized the experimental gene list as seeds (represented by the larger nodes in Figure 3B) to obtain the top-ranking neighbor genes (smaller nodes) [32,33,34]. To illustrate the involvement of the immune system, we highlighted the genes that are part the GO term “immune system process” (GO:0002376) in red. This additional analysis resulted in a larger list of the enriched GO terms, strengthening the initial ORT analysis (Table S4). The amount of redundancy and overlaps in GO terms can make the interpretation challenging, therefore we reduced the resulting GO terms using semantic uniqueness [35] and showed only the most representative terms of the affected biological pathways [36]. The reduced GO terms highlighted the importance of immune processes (three cluster of nodes), cell surface receptor signaling (two clusters), and metabolic and growth processes (Figure S4) in response to vitamin D3 supplementation.

Interestingly, the downregulation of LAIR1, a negative regulator of cytolytic functions in natural killer (NK) cells, B-cells, and T-cells, was one of the major nodes seen in our seeded-network analysis. While the immune functions seemed to be diminished with supplementation, the reduced abundance of LAIR1, in contrast, would facilitate TCR-mediated activation, since the capacity to inhibit NF-kB translocation and hematopoietic precursor differentiation to dendritic cells (46) would be decreased.

Overall, the molecular changes and cellular processes identified encompass and agree with the gene enrichment results. Major clusters were related to immune system processes such as response to cytokine and stimuli, and catabolic processes which is represented by the ubiquitin system. Our targeted transcriptomic assays captured several of the most reported genes modulated by vitamin D (Figure S5), providing confidence to our gene panel coverage, and, as well, identified putatively novel mediators of vitamin D3.

## 3. Discussion

Our study enrolled 100 vitamin D-deficient but otherwise healthy women who have received a weekly oral dose (50,000 IU) of vitamin D3 for three months. Our primary objective was to investigate the effect of vitamin D3 on targeted blood transcriptomic profile. Information on the response to vitamin D3, calculated based on the achieved levels of 25(OH)D, allowed us to investigate factors affecting an individual’s response to the treatment.

Vitamin D is well-documented to exert genomic effects via the transcription factor activity of the vitamin D receptor (VDR) on target gene promoters [37,38,39,40,41]. In addition, non-genomic effects of vitamin D include modulations of other signaling cascades, such as those of the innate and adaptive immunity, which has been reviewed [42]. Regarding modulatory effects on innate immunity, vitamin D3 has been shown to down-regulate TLR2, 4, and 9 expression in cultured monocytes from healthy volunteers [43,44]. Interestingly, this decrease was accompanied by impaired NF-kB nuclear translocation. More recently, vitamin D3 has shown to reduce TLR3, 7, and 9 gene expression in cultured PBMCs from systemic lupus erythematosus patients [45]. Our results indicated the involvement of the immune system in the response to vitamin D3 supplementation, regardless of the response groups to which the subject belongs to, particularly with the downregulation of pro-inflammatory markers, IFNγ receptor, and NF-kB pathways.

Vitamin D can act at multiple levels to modulate immune responses (reviewed in [2]), such as by reinforcing the physical barrier function of epithelial cells, maintaining a healthy gut microbiota [2,46,47], and promoting a high expression of VDR to facilitate the maturation of monocytes to phagocytic macrophages [48,49]. Vitamin D levels have also been linked to the efficiency to fight microbial infections [50], as well as protection against other infections [51,52,53] through the upregulation of CAMP and expression of the antimicrobial peptide LL-37 [54]. LL-37 is known to interact with GPCRs, receptor tyrosine kinases (RTKs), ligand-gated ion channel (LGIC), Toll-like receptors (TLRs), and can indirectly inhibit TLR4 via targeting of [55] p38.

Derived from whole blood gene expression, we found evidence of attenuation of GPCR activity and conversion of PIP3 to PIP2 by vitamin D3; speculatively, this is interpreted as either an attempt to reinforce homeostasis or to diminish signaling events from GPCR (IP3 to calcium, DAG to PKC, PI3K to possibly AKT) and preferential inhibition of immune functions [56]. This supports the notion that innate immunity can be affected by an increase in circulating 25(OH)D levels due to supplementation.

Immunoregulatory effects of vitamin D on immune cells have been recently reviewed [57]. From our results, we have pinpointed the putative facilitation of TCR-mediated activation via downregulation of LAIR1. Interestingly, in the public dataset GSE94138, HLADRB4, a class II major histocompatibility complex, is the most upregulated gene (2.28 FC) after three months of vitamin D supplementation (data not shown). Together with the decreased expression of LAIR1, this finding suggests the priming of antigen presentation capacity (possibly in monocyte/macrophages) and facilitation of TCR activation. On the other hand, active vitamin D can inhibit DC differentiation and maturation, modulate DC-derived cytokine and chemokine expression [58], and promote the creation of tolerogenic myeloid DCs, leading to decreased Th1 cell development and increased Treg [58,59,60,61,62,63]. Suppression of the effector immune response is known to be partly mediated by the downregulation of NF-kB, a central mediator of pro-inflammatory responses [42]. In line with the literature, we observed a consistent downregulation of genes involved in NF-kB pathways (i.e., NLRC5, CDC34, HERC3, IKBKG) in Phase II, supporting an innate-modulated inhibition of adaptive immunity in the presence of increased vitamin D3 levels. Considering the decrease in TLRs expression mentioned above, vitamin D3 supplementation might have particularly beneficial use in patients with chronic and/or auto-inflammatory diseases. We have also reported an increased abundance of E4F1 following vitamin D3 supplementation, which highlights the putative role of E4F1 as an important modulator of cell fate: Proliferation or growth arrest [64,65,66]. However, in the context of our study, the involvement of E4F1 in the modulation of T cell populations would require a more exhaustive phenotypic characterization of blood cells.

The changes observed in the IFNG pathway could be indicative of specific downregulations of immune responses by vitamin D3, exerted via the decrease in cell-mediated immunity and attenuation of T cells immune responses. However, a multitude of interferon stimulated genes (ISGs) exist with redundant/complementary functions which were not fully captured by our targeted panel. An exploration of a public dataset (GSE94138) with similar treatment regimen and duration showed that several interferon-induced or related genes, such as IFI44L (−1.89 FC), IFIT1 (−1.76 FC), IFI44 (−1.62 FC), IFIT3 (−1.51 FC), and IFI6 (−1.46 FC), were down-regulated after a three-month vitamin D supplementation. A more exhaustive investigation of the type 2 IFN-mediated ISGs expression following vitamin D3 supplementation would illuminate on the specific spectrum of activations and attenuations.

Relying on circulating vitamin D target levels alone, i.e., as a cutoff rather that an average, is unlikely to be sufficient in determining the actual vitamin D requirement of an healthy individual [67]. The response threshold applied in the study does not necessarily translate to an adverse effect for the non-responders—a longer supplementation period may be required for those individuals in order for their circulating vitamin D levels to suffice our threshold of 20 ng/mL. Factors such as the gut microbiota composition [12] can also affect how vitamin D is absorbed/processed [68,69]. As well, vitamin D in circulation (i.e., bioavailability) may be affected by the metabolic composition of blood [70]. Additionally, immune responses can be affected by multiple environmental factors [71,72] and may account for some differences seen in the effects of vitamin D3 on the blood immuno-transcriptomic. Thus, labelling individuals as R or NR based on the achieved levels of vitamin D may not be the most appropriate way to determine the biological effectiveness of the supplementation regimen. Indeed, we have observed drastic transcriptomic effects in both response groups induced by vitamin D3 supplementation (Figure 1).

## 4. Materials and Methods

### 4.1. Study Design and Sample Collection

**Figure 4:**
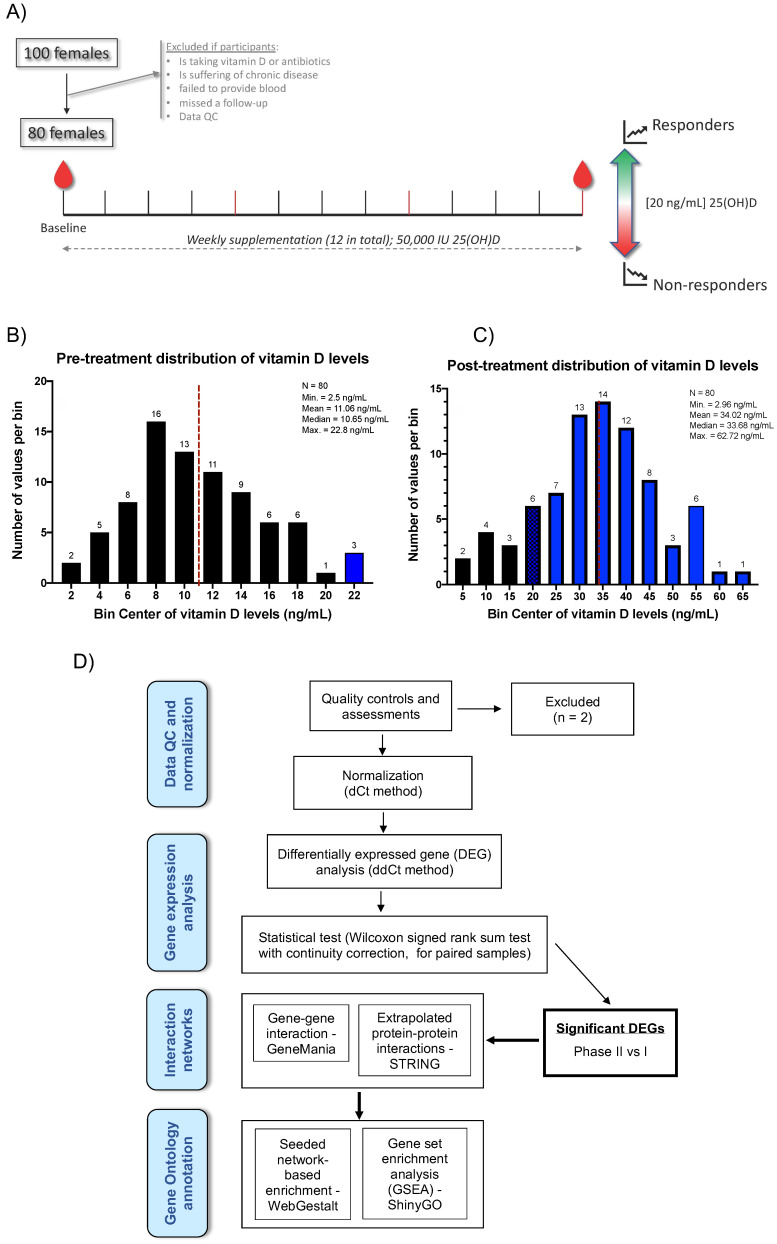
Study design and vitamin D levels of our cohort. (**A**) A total of 80 from the 100 recruited subjects were included in the study. Each participant received a weekly oral supplementation of 50,000 IU of vitamin D3 (i.e., 25(OH)D). The change in vitamin D levels after three months of supplementation was used to classify each participant as either a responder or non-responder. (**B**) Distribution of vitamin D levels among the participants prior to supplementation. The colors indicate the pre-supplementation status based on 25(OH)D concentration: Black bars represent deficient individuals (≤20 ng/mL 25(OH)D) and blue bars sufficient individuals (≥21 and ≤30 ng/mL 25(OH)D). (**C**) Distribution of vitamin D levels among participants after vitamin D3 supplementation. The colors indicate the post-supplementation status based on 25(OH)D concentration: Black bars represent deficient individuals (≤20 ng/mL 25(OH)D) and blue bars sufficient individuals (≥21 and ≤30 ng/mL 25(OH)D). The fourth bar, in blue with black grid pattern and bin center of 20, contains 1 individual with vitamin D level of 19.36 ng/mL, belonging to the deficient group; the other five belong to the sufficient group. (**D**) Flow diagram of our analysis pipeline. Fold-changes and statistical tests were calculated from the comparison of normalized gene expression of phase II vs. I (i.e., post vs. pre vitamin D supplementation). The significant differentially expressed genes were submitted to network and gene ontology analyses using the tools indicated.

The study was approved by Qatar University’s Institutional Review Board (QU-IRB; 531-A/15) and by Sidra Medicine IRB (1705010938). One hundred healthy female students at Qatar University (Doha, Qatar) were enrolled in the study. All participants underwent physical examination and submitted written informed consents for study participation and data publication. Participants were asked to complete a pre-structured questionnaire that included present and past medical history, exposure to sunlight, and other relevant details. Following confirmation of vitamin D deficiency, each subject was prescribed a weekly 50,000 IU vitamin D for three months. To ensure compliance and that the subjects were taking the supplementation, emails were sent to each of them and, additionally, participants were reached via phone calls. Fasting peripheral blood (~4 mL) was drawn at pre (Phase I) and post (Phase II) 25(OH)D (interchangeable with vitamin D3) supplementation by a licensed nurse (Figure 4A). For gene expression analysis, whole blood was collected in PaxGene Blood RNA tubes (BD Biosciences, USA). All samples were stored in −80 °C until analysis. Serum 25(OH)D was measured using the DIAsource 25OH vitamin D Total ELISA 90′ Kit (DIAsource ImmunoAssays SA, Belgium). At the end of the supplementation (Phase II), participants were classified as either responder (R; those who achieved [25(OH)D] > 20 ng/mL) or non-responder (NR; those whose [25(OH)D] < 20 ng/mL) [73]. Enrolled subjects were excluded if they were taking additional vitamin D or antibiotics, suffering from chronic disease, missed a follow-up visit, or failed to provide complete blood samples. Participant samples were excluded from analyses if they failed to pass qPCR quality assessment. A total of 80 subjects were included in the study (Figure S6).

### 4.2. Transcriptome Fingerprint Assay—A Reduced Blood Transcriptomic Panel

**Figure 5:**
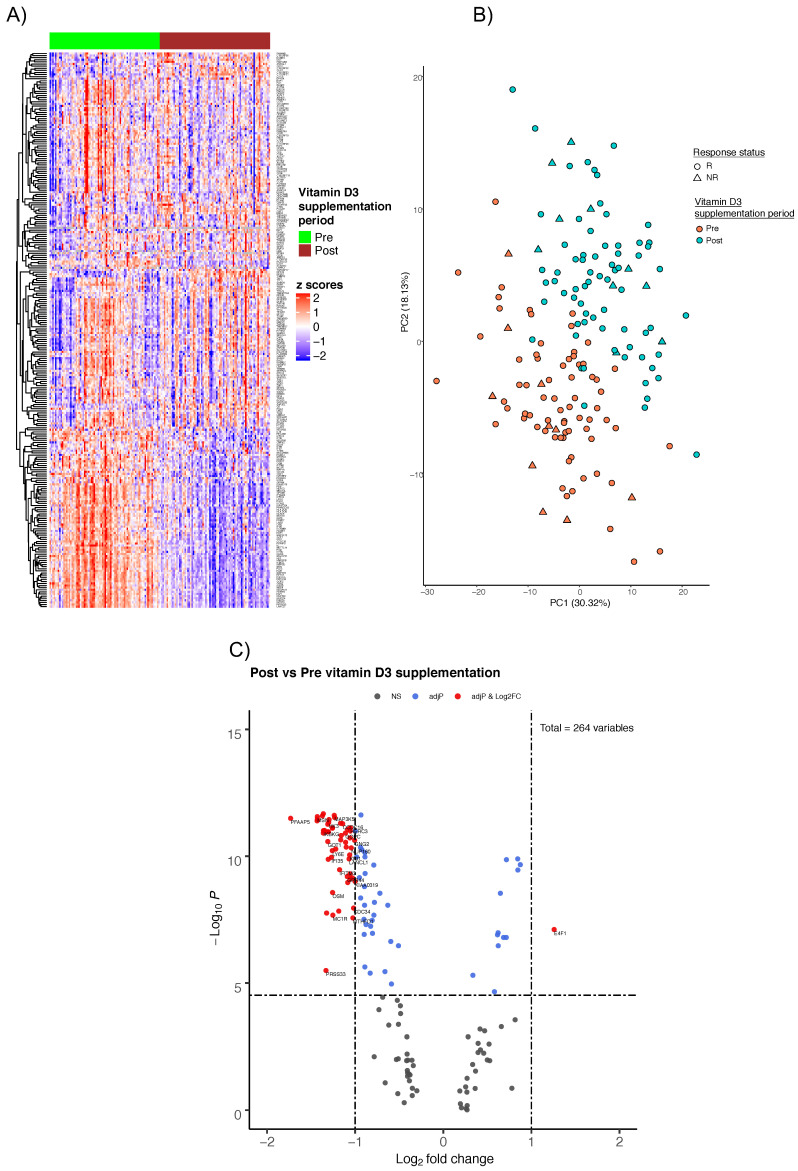
Gene expression profile and differential analysis pre and post vitamin D3 supplementation. (**A**) Heatmap representation of normalized gene expression profiles ordered by supplementation phases/periods (i.e., pre and post vitamin D3 supplementation periods). The rows represent the targeted gene panel (264 genes) and the columns represent the pre and post vitamin D3 supplementation periods of the 80 study participants (total 160 columns). (**B**) Principal component analysis overlapped with the participants’ response to vitamin D supplementation (see METHODS for details). Supplementation periods are depicted by color and the response status by shape as described in the legend. (**C**) Volcano plot representation of the differentially expressed genes between supplementation periods; Log_2_ fold-change and -Log_10_*p*-value are expressed on the x- and y-axis, respectively. Genes that are non-significantly expressed (NS) are depicted as black dots. Blue dots correspond to genes that have a Log_2_ fold-change between −1 and 1, but with significant *p*-values. Red dots correspond to genes that have a fold-change smaller than −1 or greater than 1 and significant *p*-values. Statistical comparison of gene expression between pre and post periods was performed using Wilcoxon Signed Rank Test with Bonferroni correction. Significance was assigned if adjusted *p*-value < 3 × 10^−5^ and is indicated by the horizontal dashed black line. The vertical dashed black line indicates a Log_2_ FC of 1 in gene expression.

The transcriptome fingerprint assay has been previously described [74,75,76] and is composed of 382 modules, each formed by a group of co-expressed genes across a reference collection of 16 blood transcriptome datasets of health and disease cohorts. The modules were previously annotated using the top three most significant MeSH terms (PubMed) association using Literature Lab (Acumenta Biotech) [77]. A detailed description of the method and analytical approach are publicly available online [78]. The reduced blood transcriptomic panel is a representative subset of the original, which best reflects the variability seen across the source data. In brief, the modules were partitioned with Hartigan’s K-means algorithm in JUMP [12] to reduce granularity and to determine the appropriate number of subgroups. If a subgroup did not contain at least one module in one of the 16 diseases showing at least 25% of genes up- or down-regulated, it was excluded from selection. The module closest to the mean vector of each subgroup was selected, resulting in 66 representative modules. The composite genes were then ranked according to the distance to the group means, and the four highest-ranking genes symbols were selected to represent the reduced module (Figure 5). A total of 264 genes and eight housing keeping genes (DOCK2, EEF1A1, FAM105B, FTL, MYL6, MYL12B, RPS10, and RPS25) were included in dynamic arrays for gene profiling.

### 4.3. Gene Expression Profiling and Statistical Analysis

Total RNA from whole blood was extracted using the QIAsymphony PAXgene Blood RNA Kit (Qiagen, Germany), following the manufacturer’s instruction. RNA quantity and purity were checked by NanoDrop (Thermo Fisher Scientific, Waltham, MA, USA, UES) and quality was determined on the Fragment Analyzer System (Agilent, Santa Clara, CA, USA). Reverse transcription was performed on the isolated RNA using Fluidigm cDNA synthesis kit (Fluidigm Corporation, South San Francisco, CA, USA), and gene expression was determined by parallel quantitative PCR using Fluidigm BioMark HD platform (Fluidigm Corporation). Transcript specific assays were designed and ordered through the Fluidigm D3 Assay Design tool (d3.fluidigm.com, accessed on 13 April 2021). The PCR reaction conditions were as follows: Denaturation at 95 °C for 10 min, followed by 30 cycles at 95 °C for 15 s and 60 °C for 1 min according to the protocol for gene expression. Three dynamic arrays, each containing 88 genes of interest and the eight housekeeping genes, were run for each sample. Distilled water was used as negative control and pooled RNA isolated from healthy individuals was used as internal batch control. To verify primer specificities, melting curves were generated at the end of each PCR reaction. Representative amplification plots and melt curves for ten genes among the DEGs identified, including four previously reported vitamin D-responsive genes [79], are shown in Figure S7.

Fluidigm Real Time PCR software (Fluidigm Corporation) was used to merge the technical runs and threshold cycle (Ct) values were processed using Partek Genomic Suite (version 7.18; Partek, USA); see details in Supplementary Materials. Genes with Ct values beyond the detectable range were set as missing values for downstream analyses. Raw data were assessed for inter- and intra- sample variation, and our internal control samples were validated. Samples not passing our QC criteria were excluded. Each gene was then normalized to a validated house-keeping gene pool and reported as dCt (Additional File 1) and transformed to −dCt for principal component (PCA) and hierarchical clustering analyses. Fold-changes and statistical tests were calculated from the comparison of normalized gene expression of phase II vs. I (i.e., post vs. pre vitamin D supplementation). Unless otherwise stated, data analyses were performed in R. Missing values were estimated using missMDA [80] prior to PCA. Euclidean distances and the complete linkage method were used for the hierarchical clustering analysis. Differentially expressed genes (DEGs) between Phase II and I were determined using a paired non-parametric two sample Wilcoxon test (aka Wilcoxon Signed Rank test) with the following criteria to denote significance: FC > 2 and adjusted *p*-value ≤ 0.01.

### 4.4. Networks, Gene Ontology, and Annotation

Gene–gene and predicted protein–protein interaction networks of the DEGs were generated with GeneMania [81] and STRING [82], respectively. For STRING, a minimum interaction score of 0.4 and FDR < 0.05 were applied. The term tree and gene ontology (GO) network were generated with ShinyGO v0.61 [31]; a FDR < 0.05 was applied. Network-based enrichment analysis was performed by the R-based web tool WebGestalt [32] which utilizes Gene2Net; a *p*-value < 0.001 was applied. Gene2Net allows users to expand one or multiple genes into a sub-network based on the selected networks, performs guilt-by-association analysis based on the GO database and compares the different sub-networks and corresponding functions. The top 10 neighbors were selected based on the probability of random walk method. All seeds and top-ranking neighbors in the expanded sub-network were able to enrich to 366 GO biological pathway categories. Semantic reduction and visualization of the enriched GO terms was generated using REVIGO [36], at a dispensability <0.5.

### 4.5. Literature Lab^TM^ Gene Retriever

PubMed IDs (PMID) obtained from the PubMed query below were submitted to Literature Lab^TM^, (Acumenta Biotech, acumenta.com) Gene Retriever application [77]. We identified 1300 genes with 608 genes associated with more than one PMID published up to 12 August 2020. Query: “vitamin d” [MeSH Terms] or “ergocalciferols” [MeSH Terms] or “vitamin D” [All Fields] or “calcifediol” [All Fields] or “25-hydroxycholecalciferol” [All Fields] or “25-hydroxyvitamin D” [All Fields] and (“immunology” [Subheading] or “immunology” [All Fields] or “allergy and immunology” [MeSH Terms] or “immune response” [All Fields] or “immune regulation” [All Fields] or “immunity” [MeSH Terms] or “immunity” [All Fields]) and “humans” [MeSH Terms].

## 5. Conclusions

**Figure 6:**
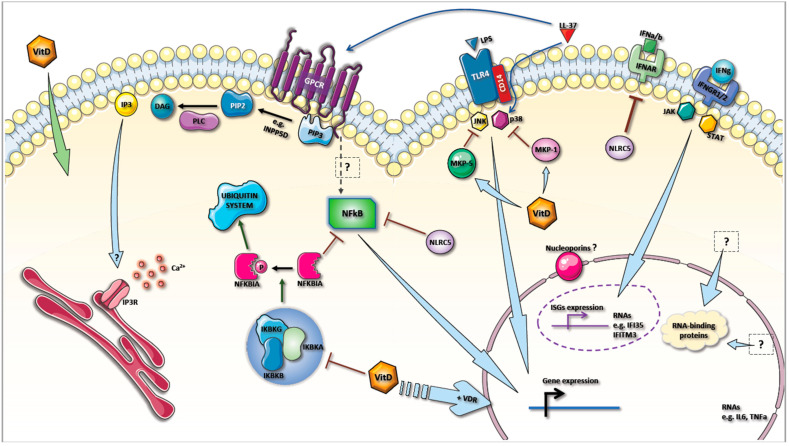
Schematic summary of findings. Interpretation of the differentially expressed genes and the enriched biological process identified several interconnected cellular signaling pathways. Our findings highlight roles for vitamin D3 in modulating: (1) Cell surface receptor signaling, including G-coupled receptors and phosphatidylinositol pathways; (2) ubiquitin system; and (3) IFNg receptor and LPS receptors (TLR4, CD14) signaling, possibly involved in mediating the regulation of cytokine production. Arrow indicates action or pathway; blunted arrow indicates inhibition; dashed arrow indicates unspecified action(s) or pathway(s); “?” indicates unknown mediator(s) or action(s).

Our study provides a blood transcriptomic resource of the changes that occurred with a three-month weekly course of vitamin D3 supplementation. We summarized our findings in a schematic representing the DEGs and biological processed altered by vitamin D3 (Figure 6). The information could be augmented by a larger cohort size and/or untargeted transcriptomic approach. Nonetheless, we observed downregulation of pro-inflammatory pathways (e.g., cytokine signaling pathways) upon vitamin D3 supplementation, acting via alterations at the levels of TLR4/CD14 and IFN receptors as well as regulating the activity of NF-kB. The PIP3 to IP3 pathway was also negatively regulated, and this represents a possible implication on calcium metabolism although it was not the most predominant perturbation observed. Interestingly, our results indicated that the ubiquitin system may play an important role in mediating vitamin D-dependent regulation of NF-kB. Furthermore, our results point to a reduction in membrane receptor-mediated signaling, but also to facilitation of TCR-mediated activation via a potentiated antigen presentation by monocytes/macrophages. Pathological conditions or treatment affecting the pathways we identified may warrant closer examination of the effect of Vitamin D in clinical settings. Indeed, many therapeutic avenues are being developed for diseases such as cancer, suggesting that the lack of response in some individual might represent a dysregulated non-classical vitamin D pathway.

## Data Availability

The datasets presented in this study can be found in the Gene Expression Omnibus (GEO) via the National Center for Biotechnology Information (NLM, NIH). The accession number is GSE157939. Access will be made publicly available upon publication of the article

## Supplementary Materials

The following are available online at https://www.mdpi.com/article/10.3390/ijms22095041/s1.

## Abbreviations

Aside from official gene names, the following abbreviations were used in-text and legends:

BMI	Body-mass index
CAMP	cathelicidin antimicrobial peptide
CD14	cluster of differentiation 14
COVID-19	Coronavirus disease
Ct, dCt, ddCt	Threshold cycle, normalized Ct, differential dCt
CYP27B1	cytochrome P450 family 27 subfamily B member 1
DC	Dendritic cell
DEGs	Differentially expressed genes
GO	Gene Ontology
GPCR	G protein-coupled receptors
IFN	Interferon
IL6	Interleukin 6
LGIC	ligand-gated ion channel
MAP	Mitogen-activated protein
MeSH terms	Medical subject headings terms
NF-kB	Nuclear factor kappa B subunit 1
NR	Non responders
ORT	Over representation test
PCA	Principal component analysis
PCR	Polymerase chain reaction
PI3K	Phospho-inositol-3 kinase
PIP2	Phosphatidylinositol (4,5)-bisphosphate
PIP3	Phosphatidylinositol (3,4,5)-trisphosphate
PMID	PubMed ID
R	Responders
ROS	Reactive oxygen species
RTKs	Receptor tyrosine kinases
SARS-CoV-2	severe acute respiratory syndrome coronavirus 2
Th1 cell	T helper type 1 cell
TFA	Transcriptomic Fingerprint Assay
TLR	Toll-like receptor
TNF	Tumor necrosis factor
Treg cell	T regulatory cell
VDR	Vitamin D receptor
VDRE	Vitamin D response element
Vitamin D3	25-(OH)D or cholecalciferol
