# Supplementary material for: Immunomodulatory Effects of Vitamin D Supplementation in a Deficient Population
Source: Int J Mol Sci. 2021 May 10;22(9):5041. doi: 10.3390/ijms22095041 (PMC8126205; doi:10.3390/ijms22095041)
Supplement: Supplementary file 1 [file ijms-22-05041-s001.zip › ijms-1159989-supplementary.pdf]

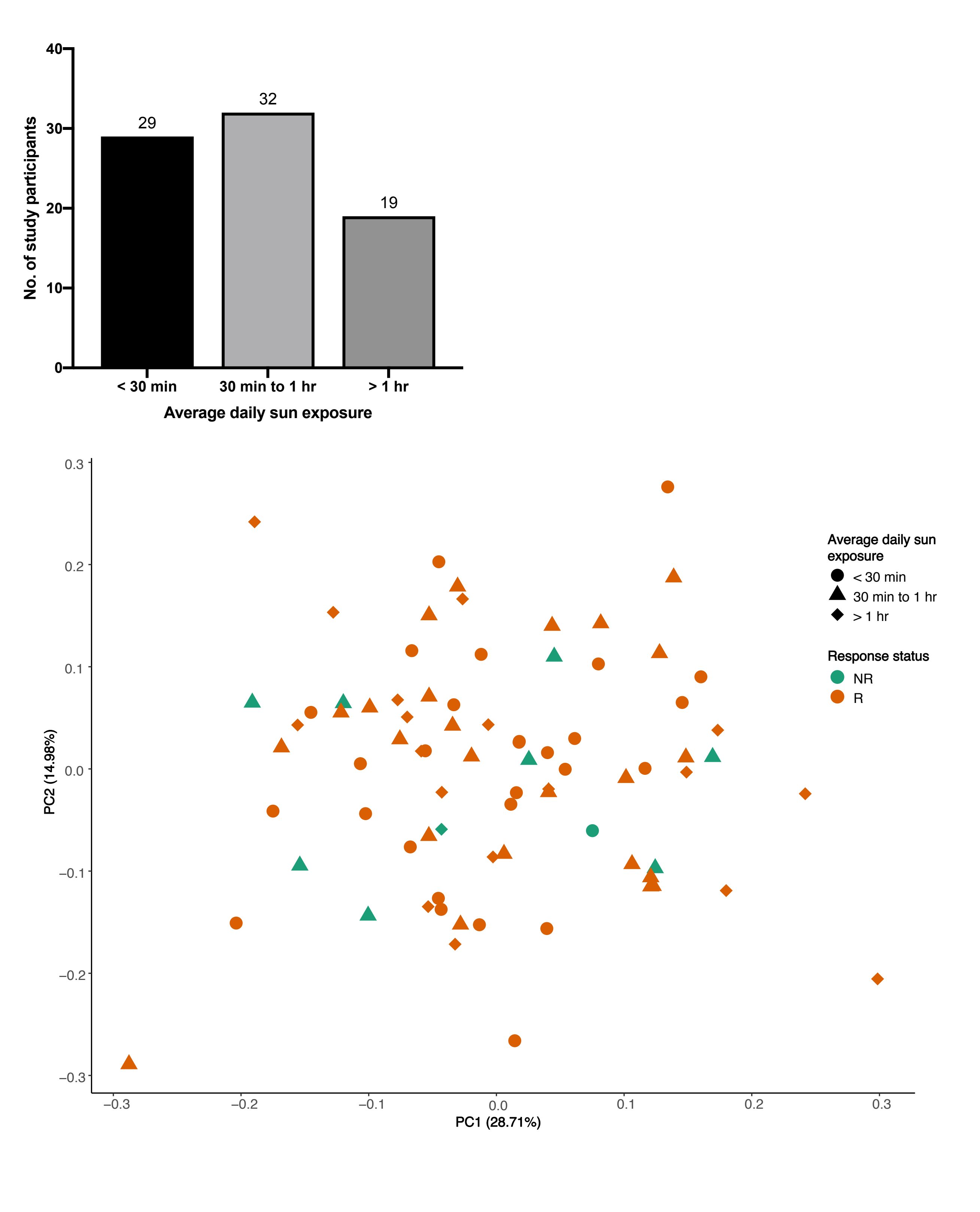

Suppl. Figure 1. **Average daily sun exposure and its effect on gene expression**. Principal component analysis shows that sun exposure group does not results in separation of samples nor influences response status. Circles = “less than 1/2 hr”; triangles = “1/2 to 1 hr”; and lozenges = “more than 1 hr”. For reference, the response status to vitamin D supplementation is indicated: green = non-responders (NR) and orange = responders (R).

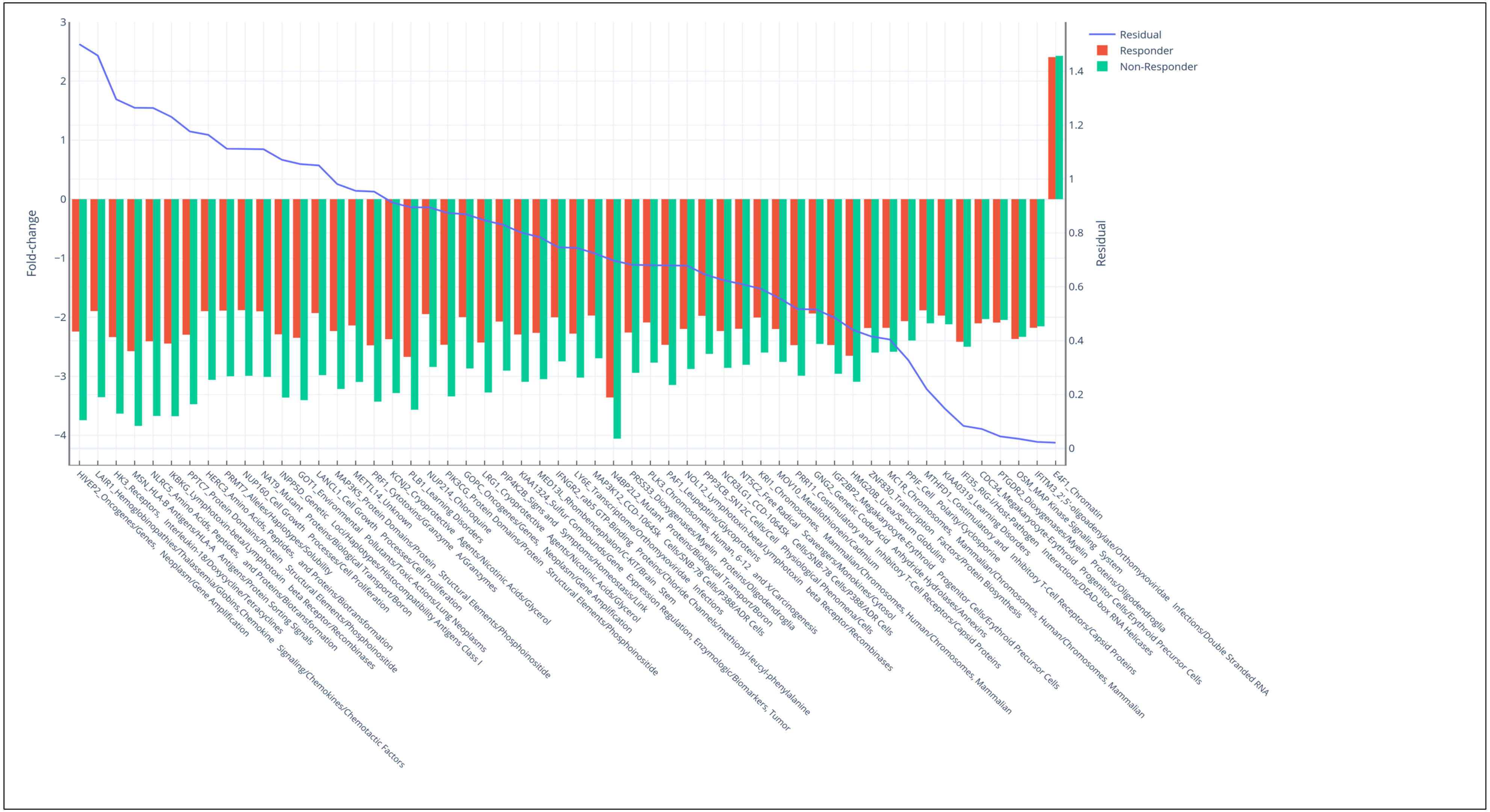

Suppl. Figure 2. **The extent of downregulation in the non-responders is more pronounced compared that of the responders**. Comparison of the fold-changes (FC) between responders (R) and non-responders (NR) for the identified significant differentially expressed genes (DEGs; n = 54) between Phase II and I. FC is shown of the left y-axis and residual (i.e. difference between FC of R and NR) on the right y-axis. The 54 DEGs and their respective module title are shown on the x-axis. The difference between NR and R in terms of gene expressions did not reach significance.

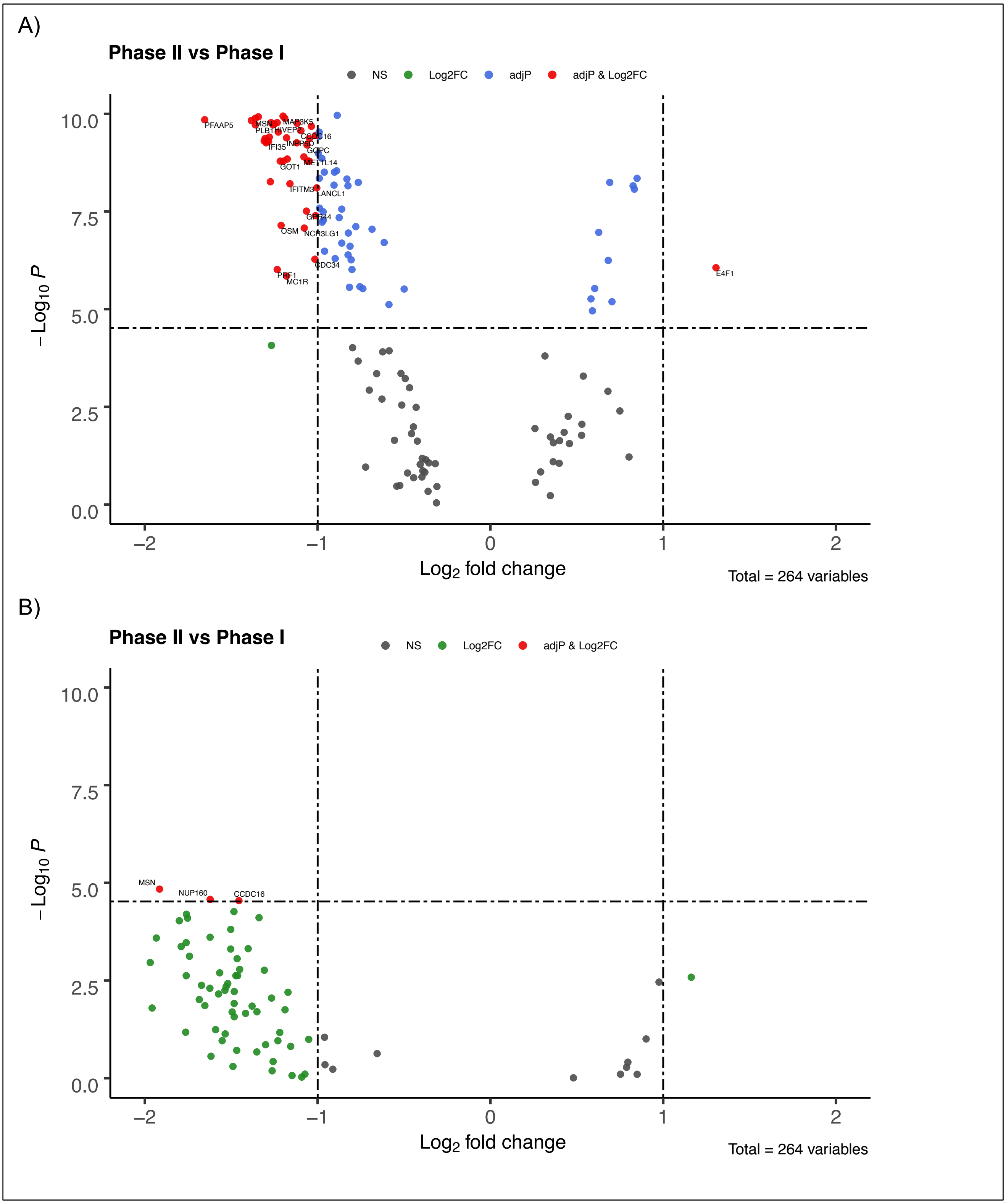

Suppl. Figure 3. Differential gene expression in the responder and non-responder subsets. Volcano plot representation of the differentially expressed genes between Phase II and I in the responders (**A**) and non-responders (**B**) subset of individuals. Log2 fold-change and -Log10P-value are expressed on the x- and y-axis, respectively. Genes non-significantly expressed (NS) are depicted by black dots. Blue dots correspond to genes that have Log2 fold-change between -1 and 1, but with significant p-values. Red dots correspond to genes that have fold-change smaller than -1 or greater than 1 and significant p-values. Statistical comparison of gene expression between Phases was performed Wilcoxon Signed Rank test with Bonferroni correction at adjusted $p < 3E-5$. The horizontal dashed black line indicates the adjusted p-value cut-off and the vertical dashed black line indicates a Log2 FC of 1 in gene expression.

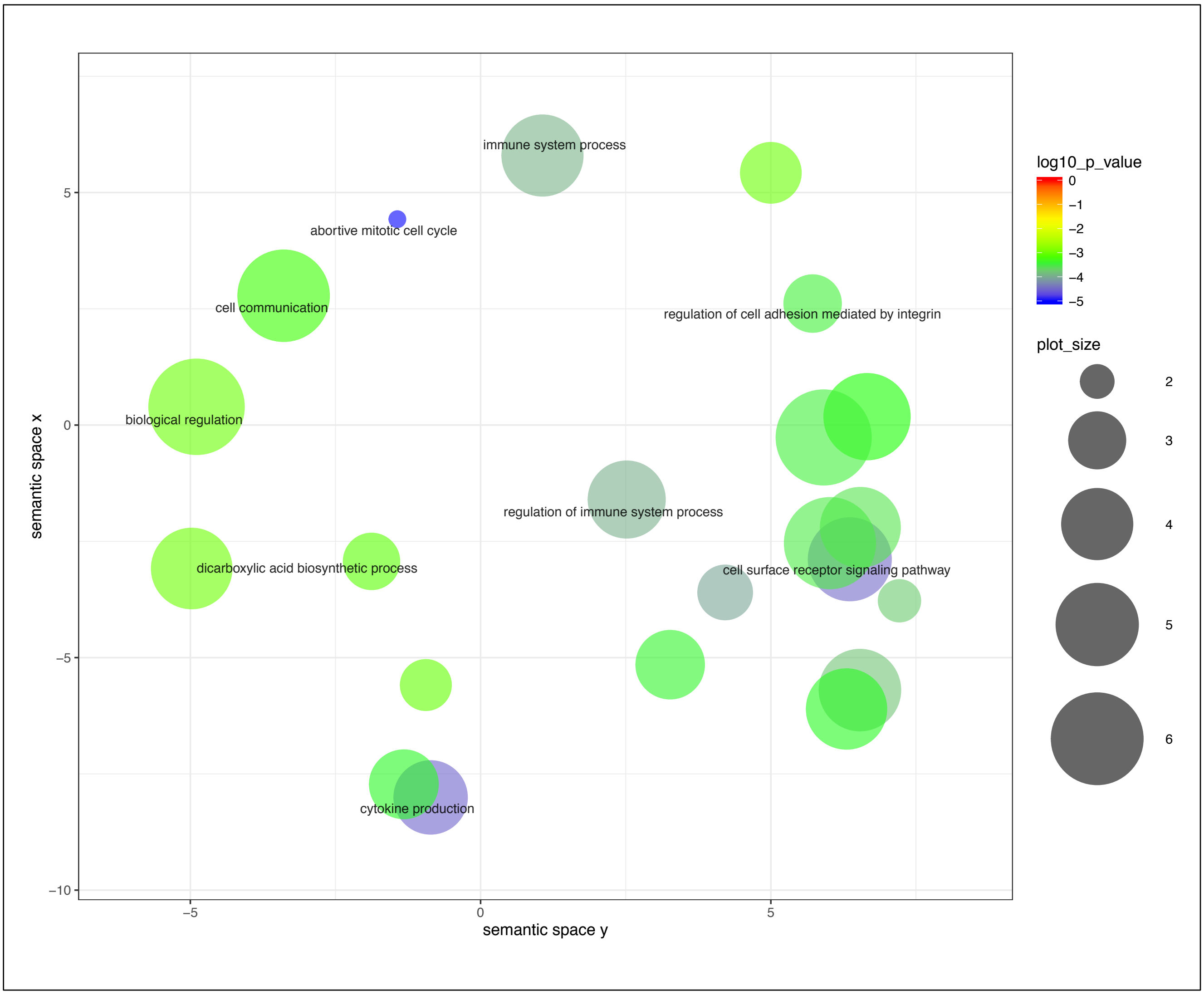

Suppl. Figure 4. Simplification and visualization of the enriched gene ontology (GO) terms with p-value < 0.001. The similarity plot shows the GO terms with enrichment p-value < 0.001 separated according to semantic similarity (generated with REVIGO, see METHODS). Nodes with labels represent the most indispensable terms and form representative clusters for term with similar semantic.

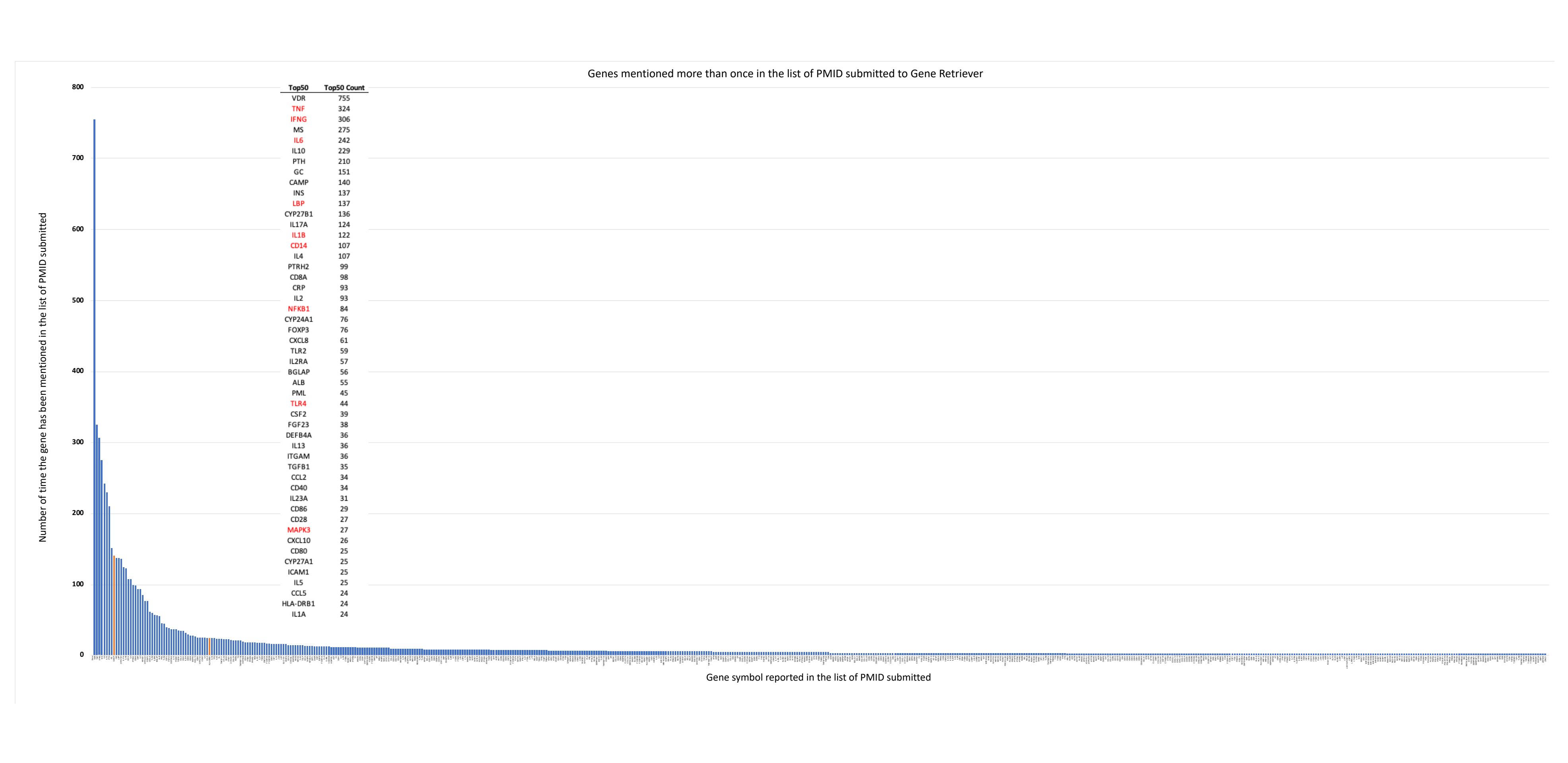

Suppl. Figure 5. **Extraction of the genes associated with vitamin D from the NCBI PubMed database**. 4065 abstracts/articles were returned from the PubMed literature search using the following search strategy: "vitamin d"[MeSH Terms] OR "ergocalciferols"[MeSH Terms] OR "vitamin D"[All Fields] OR "calcifediol"[All Fields] OR "25-hydroxycholecalciferol"[All Fields] OR "25-hydroxyvitamin D"[All Fields] AND ("immunology"[Subheading] OR "immunology"[All Fields] OR "allergy and immunology"[MeSH Terms] OR "immune response"[All Fields] OR "immune regulation"[All Fields] OR "immunity"[MeSH Terms] OR "immunity"[All Fields]) AND "humans"[MeSH Terms]. Gene Retriever (**Literature Lab™**, Acumenta Biotech, https://www.acumenta.com) was used to obtain all the genes linked/tagged with each abstract. The top 10 and 50 gene are highlighted in the insert inside the plot. The plot shows the number of times the gene has been mentioned in the list of PMID submitted (y-axis) and the gene symbol reported in the list of PMID submitted. A total of 1300 genes are ranked by the number of reporting on the x-axis. Major genes associated with the immune regulation of vitamin D which were described in the main text are highlighted in red.

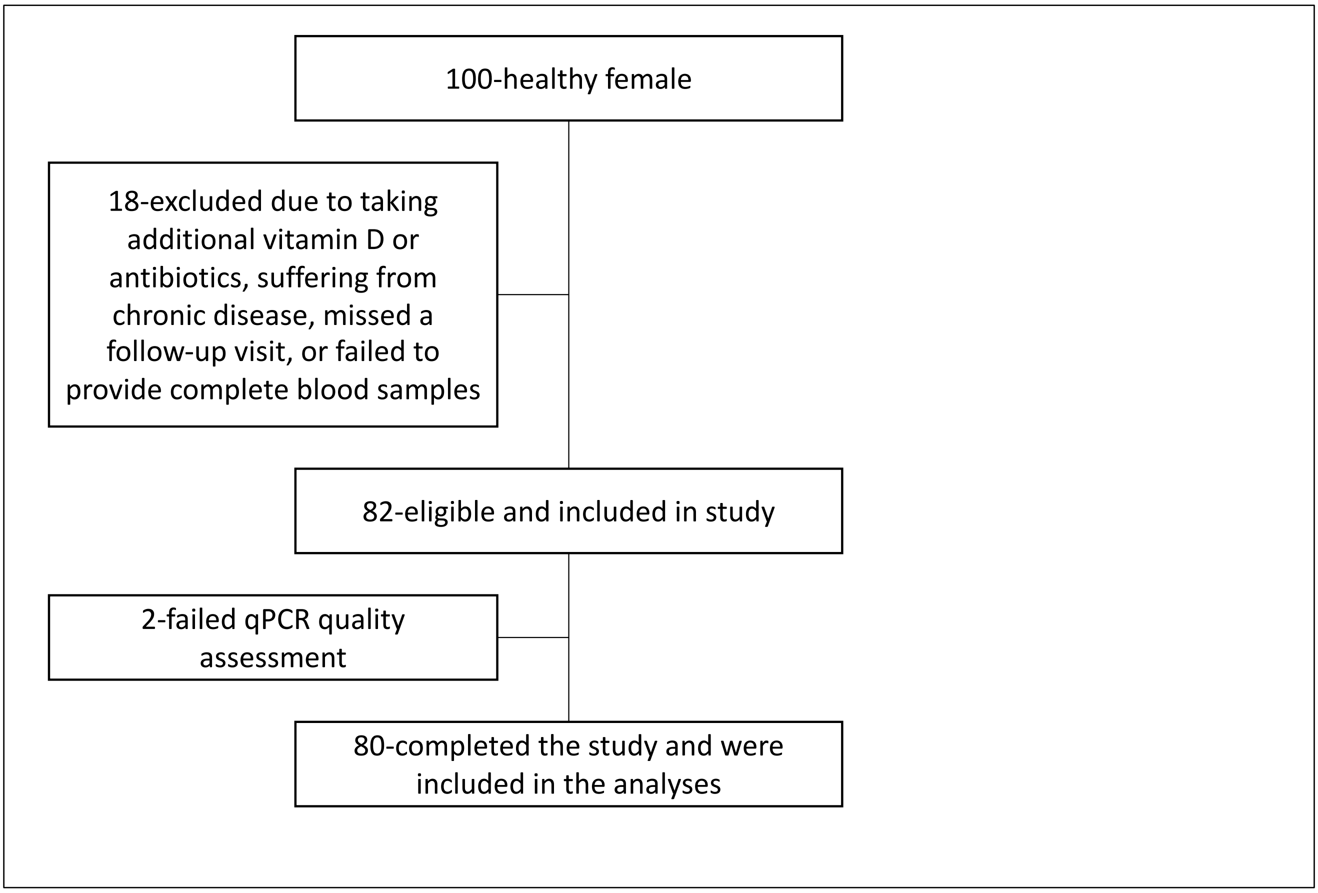

Suppl. Figure 6. **Participant flow chart**. Recruitment and retentions of subjects in the study and downstream analyses.

LAIR1 gene (Amplification Plot)

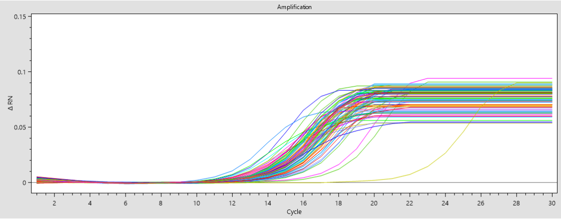

LAIR1 gene (Melting Curve)

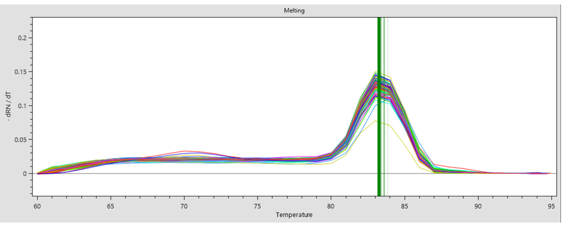

HIVEP2 gene (Amplification Plot)

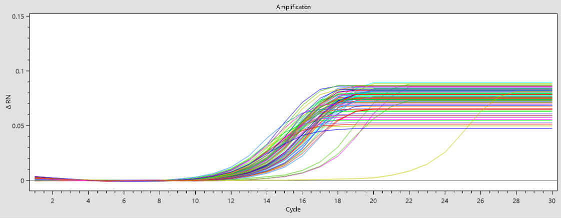

HIVEP2 gene (Melting Curve)

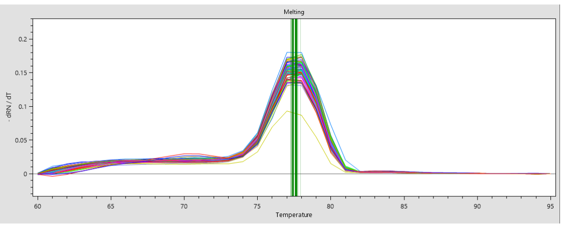

KCNJ2 gene (Amplification Plot)

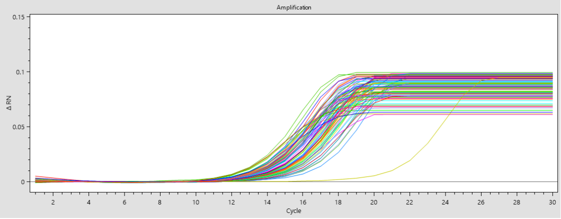

KCNJ2 gene (Melting Curve)

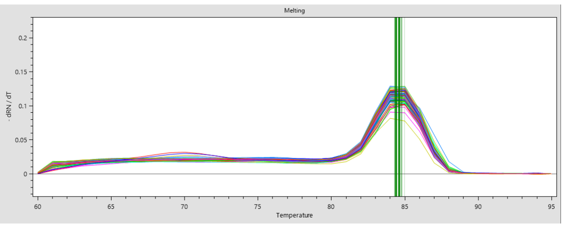

KIAA1324 gene (Amplification Plot)

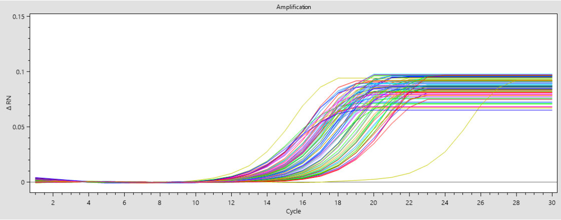

KIAA1324 gene (Melting Curve)

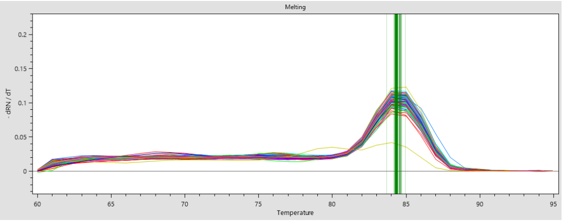

PAF1 gene (Amplification Plot)

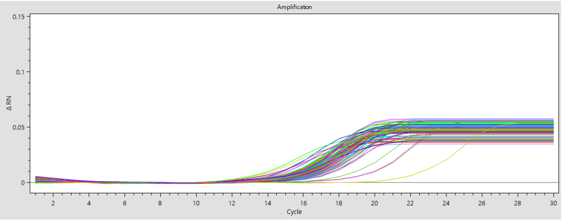

PAF1 gene (Melting Curve)

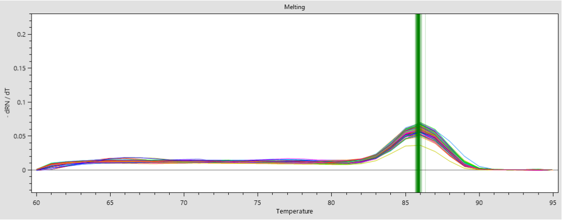

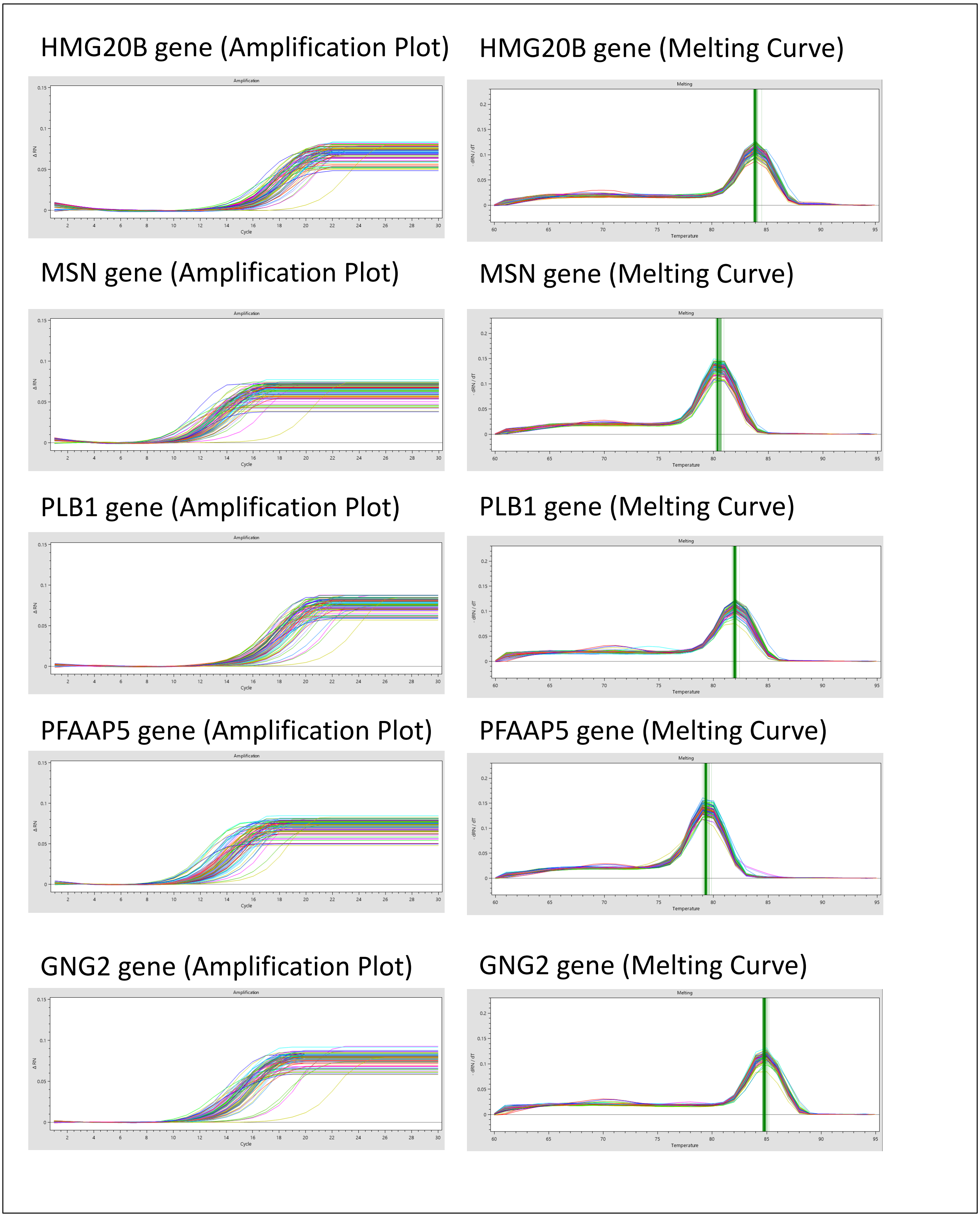

Suppl. Figure 7. **Representative amplification and melt curves**. LAIR1, HIVEP2, KCNJ2, KIAA1324, which were among the 54 DEGs we identified, have been reported as major downregulated genes following daily 10000 IU vit d for 6 months in a recent randomized double-blind clinical trial (PMID: 31776371). PAF1, HMG20B, MSN, PLB1, PFAAP5, and GNG2 are among the top DEGS identified in this study.
